# Antiandrogen and Antimicrobial Aspects of Coordination Compounds of Palladium(II), Platinum(II) and Lead(II)

**DOI:** 10.1155/MBD.2001.149

**Published:** 2001

**Authors:** R. V. Singh, S. C. Joshi, Shalini Kulshrestha, Pooja Nagpal, Anil Bansal

**Affiliations:** 1 Department of Chemistry University of Rajasthan Jaipur 302004 India; 2 Department of Zoology University of Rajasthan Jaipur 302004 India

## Abstract

Synthesis, characterization and antimicrobial activities of an interesting class of biologically potent macrocyclic complexes have been carried out. All the complexes have been evaluated for their antimicrobial effects on different species of pathogenic fungi and bacteria. The testicular sperm density, testicular sperm morphology, sperm motility, density of cauda epididymal spermatozoa and fertility in mating trails and biochemical parameters of reproductive organs have been examined and discussed. The resulting biologically active [M(MaL^n^)(R_2_)]Cl_2_ and [Pb(MaL^n^)(R_2_)X_2_] (where, M = Pd^II^ or Pt^II^ and X = Cl or NO_3_) type of complexes have been synthesized by the reactions of macrocyclic ligands (MaL^n^) with metal salts and different diamines in 1:1:1 molar ratio in methanol. Initially the complexes were characterized by elemental analyses, molecular weight determinations and conductivity measurements. The mode of bonding was established on the basis of IR, ^1^H NMR, ^13^C NMR, ^195^Pt NMR, ^207^Pb NMR, XRD and electronic spectral studies. The macrocyclic ligand coordinates through the four azomethine nitrogen atoms which are bridged by benzil moieties. IR spectra suggest that the pyridine nitrogen is not coordinating. The palladium and platinum complexes exhibit tetracoordinated square-planar geometry, whereas a hexacoordinated octahedral geometry is suggested for lead complexes.


### ANTIANDROGEN AND ANTIMICROBIAL ASPECTS OF COORDINATION COMPOUNDS OF PALLADIUM(II), PLATINUM(II) AND LEAD(II)

R.V. Singh*[1], S.C. Joshi[2], Shalini Kulshrestha[1], Pooja Nagpal[1] and Anil Bansal[1]

[1]Department of Chemistry, [2]Department of Zoology, University of Rajasthan, Jaipur-302004, India
<kudiwal@datainfosys.net> Fax : +91-141-519221

### ABSTRACT

Synthesis, characterization and antimicrobial activities of an interesting class of biologically potent macrocyclic complexes have been carried out. All the complexes have been evaluated for their antimicrobial effects on different species of pathogenic fungi and bacteria. The testicular sperm density, testicular sperm morphology, sperm motility, density of cauda epididymal spermatozoa and fertility in mating trails and biochemical parameters of reproductive organs have been examined and discussed. The resulting biologically active $[M(MaL^n)(R_2)]Cl_2$ and $[Pb(MaL^n)(R_2)X_2]$ (where, M = $Pd^{II}$ or $Pt^{II}$ and X = Cl or $NO_3$) type of complexes have been synthesized by the reactions of macrocyclic ligands ($MaL^n$) with metal salts and different diamines in 1:1:1 molar ratio in methanol. Initially the complexes were characterized by elemental analyses, molecular weight determinations and conductivity measurements. The mode of bonding was established on the basis of IR, $^1H$ NMR, $^{13}C$ NMR, $^{195}Pt$ NMR, $^{207}Pb$ NMR, XRD and electronic spectral studies. The macrocyclic ligand coordinates through the four azomethine nitrogen atoms which are bridged by benzil moieties. IR spectra suggest that the pyridine nitrogen is not coordinating. The palladium and platinum complexes exhibit tetracoordinated square-planar geometry, whereas a hexacoordinated octahedral geometry is suggested for lead complexes.

### INTRODUCTION

The chemistry of macrocyclic complexes has received much attention and such compounds have been extensively studied in recent years[1] . Multidentate Schiff bases have been used extensively in the preparation of various complexes of transition metals and main group elements, since such polydentate ligands provide unusual stablization of different oxidation states as well as a rigid coordination sphere for the central element. The chemistry of these ligands is specially developed for transition metals[2] because of their catalytic properties. The development of the field of bioinorganic chemistry has also been the other important factor in spurring the growing interest in complexes of macrocyclic compounds[3]. Macrocyclic ligand systems often exhibit unusual properties and some times mimic related natural macrocyclic compounds. Transition metal chelates of nitrogen donor ligands have been extensively screened for their antitumor activity. Most of them showed some activity but significant activities were exhibited by palladium and platinum complexes. In principle, coordination compounds offer a great variety of shapes and reactivities for use in drug design, the most detailed advances having been in understanding how cis-diammine dichloroplatinum(II) binds to DNA.

Macrocyclic complexes of transition metals having both oxo and aza groups in a ligand are well known for the ligands having dioxotetraaza[4] , tetraoxooctaaza[5] and tetraoxotetraaza[6] moieties which show some interesting properties and biological functions, such as being models for metalloproteins[4] . The interest in the synthesis and characterization of transition metal complexes containing a Schiff base lies in their biological and catalytic activity in many reactions[7]. Carbonyl complexes of transition metal ions, specially those of ruthenium, palladium and platinum, are important in homogenous catalysis such as carbonylation and the oxo reaction[8]. Furthermore, tetradentate Schiff base complexes are important for designing metal complexes related to synthetic and natural oxygen carriers[9].

Polyazamacrocyclic ligands, in their protonated forms, have shown to bind certain natural molecules and anions in solution[10] and in some instance, are able to catalyze these enzyme-mimetic reactions show enhanced rate factors when compared with the same process in the absence of macrocycle.

The synthesis of homodinuclear complexes for the Schiff base macrocycles was first accomplished by the condensation of 2,6-diacetylpyridine with 3,6-dioxaoctane-1,8-diamine in the presence of a $Pb^{+2}$ template. The homodinuclear complexation has stimulated interest in areas such as metalloenzymes, homogenous catalysis, electrical conductance and magnetic exchange processes. The striking structural features, diverse stereochemistry and appreciable agricultural, medicinal and industrial applications of palladium (II), platinum (II) and lead (II) complexes of biologically active nitrogen donor ligands led us to undertake systematic studies on the complexes of these metals. The synthesis of some new complexes of palladium (II), platinum (II) and lead (II) with the following new ligands and their structural elucidation, antifungal, antibacterial and antifertility activities are reported in the present paper.

Ligands used are as follows:

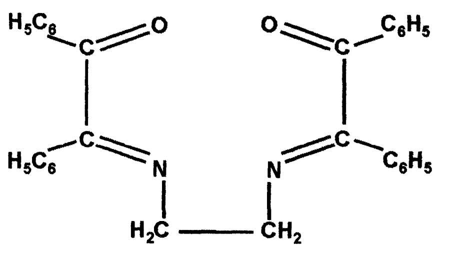

**(MaL¹)**

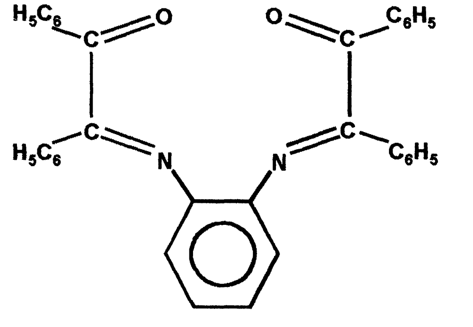

**(MaL²)**

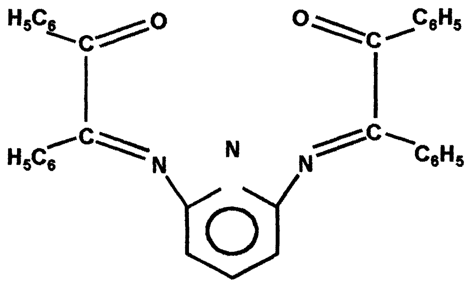

**(MaL³)**

### EXPERIMENTAL

The chemicals used were of AR grade. All the chemicals and solvents were dried and purified by standard methods.

**Preparation of the Ligands**

The said ligands were prepared by dissolving benzil in approximately 30ml of ethanol in a 100 ml round bottom flask. To this the calculated amount of diamines in ethanol (20ml) was added. The contents were refluxed for 5-7 hours on a ratio head. It was then concentrated to half of the volume. The solution was cooled and coloured crystalline compounds separated out was filtered. This was purified by recrystallization in the same solvent and dried in vacuo.

**Preparation of the Complexes**

The macrocyclic complexes of palladium(II), platinum(II) and lead(II) were prepared by the templated condensation reactions of $MaL^1$, $MaL^2$ or $MaL^3$ and 1,2-phenylenediamine or 2,6-diaminopyridine in the presence of $PdCl_2$, $PtCl_2$, $PbCl_2$ or $Pb(NO_3)_2$ in 1:1:1 molar ratio in dry methanol. The contents were refluxed for about 6-10 hours on a ratio head. After refluxing, the solution was concentrated to half of the volume by removing the solvent. The complexes crystallized on putting the contents at room temperature for overnight. They were collected, washed repeatedly with hot water then with dry and cold MeOH so as to ensure their purity and dried. The complexes were further purified by recrystallization from equimolar ratio of methanol and benzene and dried again under reduced pressure over anhydrous $CaCl_2$. The analyses of these new complexes for nitrogen, chlorine and metal agreed with the theoretical values within the limits of experimental error (Table 1).

**Analytical Methods and Physical Measurements**

Nitrogen and chlorine were determined by the Kjeldahl's and Volhard's methods, respectively[11]. Palladium and platinum were determined gravimetrically[12]. Lead was estimated as lead sulphate. Molecular weights were determined by the Rast camphor method[13] and conductivity measurements were carried out in $10^{-3}$ mol $dm^{-3}$ dimethyl formamide solution at 25°C using a Systronic type 305 conductivity bridge. Infrared spectra were recorded on a model Nicolet Megna FTIR-550 spectrophotometer in KBr pellets. The UV-visible spectra of the compounds were obtained on a Hitachi U-2000 spectrophotometer in DMSO. $^1H$ and $^{13}C$ NMR spectra were recorded on a JEOL FX 90Q spectrometer with $Me_4Si$ as an internal standard. Chemical shifts were recorded in ppm. X-ray powder diffractogram of the compounds were obtained on a Philip Model PW 1840 automatic diffractogram using Fe (Kα) target with Mg filter. The wavelength used was 1.937355 A° and the reflections from 5-65° C were recorded in almost every case. The structure was solved by the Hesse[14], Lipson[15] and It'o[16] methods. $^{207}Pb$ NMR spectra were recorded on JEOL FX200 spectrometer with $Me_4Pb$ as an external reference.

**RESULTS AND DISCUSSION**

The reactions of two molar equivalents of benzil with diamines (one molar equivalent) gives high yield of the ligands, MaL[1], MaL[2] and MaL[3].

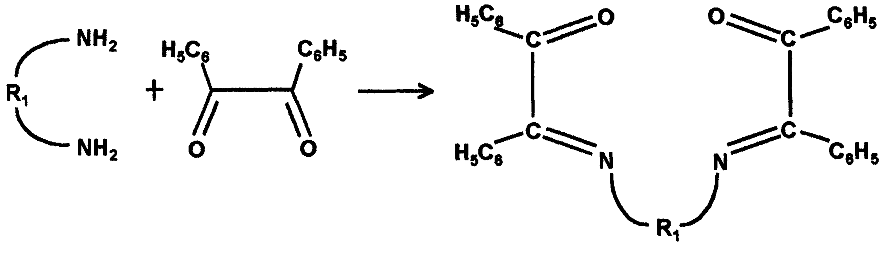

| Ligand | - | $R_1$ |
|---|---|---|
| MaL[1] | - | $-(CH_2)_2-$ |
| MaL[2] | - | 1,2-$C_6H_4$- |
| MaL[3] | - | 2,6-$C_5H_3N$ - |

The macrocyclic complexes have been synthesized by reacting $PdCl_2$, $PtCl_2$, $PbCl_2$ or $Pb(NO_3)_2$ with MaL[1], MaL[2] or MaL[3] and 1,2-phenylenediamine or 2,6-diaminopyridine in 1:1:1 molar ratio in hot and dry MeOH. These reactions are quite facile and the resulting coloured solids are stable at room temperature. These are soluble in organic solvents, DMF and DMSO without change in colour. The molar conductance of $10^{-3}$ M solutions of the compounds in anhydrous DMF lie in the range 12-30 $ohm^{-1}\ cm^2\ mol^{-1}$ indicating their non-electrolytic behaviour, while these values for the palladium(II) and platinum(II) complexes appear in the range (205-225 $ohm^{-1}\ cm^2\ mol^{-1}$) expected for 1:2 electrolytes[17] . The molecular weight determinations show them to be monomeric. The physical properties and analytical data are given in Table 1.

**Table 1. Physical Properties and Analytical Data of Ligands and their Corresponding Metal Complexes.**

| Reactants (g) | | | Product Formed (Molecular Formula) | Physical Characteristics | | | % Analysis Found (Calcd.) | | | Mol. Wt Found (Calcd.) |
|---|---|---|---|---|---|---|---|---|---|---|
| Metal Salt | Ligand | Diamines | | Molar Ratio | Colour | M.P. (°C) | N | M | Cl | |
| - | $C_{30}H_{24}N_2O_2$ (MaL[1]) | - | - | - | Reddish Yellow | 86-89 | 5.92 (6.30) | - | - | 412 (444) |
| $PtCl_2$ (0.2911) | $C_{30}H_{24}N_2O_2$ (0.4865) | $C_6H_8N_2$ (0.1183) | $C_{36}H_{28}N_4Cl_2Pt$ | 1:1:1 | Light Yellow | 138 | 6.75 (7.16) | 24.42 (24.90) | 9.44 (9.06) | 801 (783) |
| $PtCl_2$ (0.3501) | $C_{30}H_{24}N_2O_2$ (0.5850) | $C_5H_7N_3$ (0.1436) | $C_{35}H_{27}N_5Cl_2Pt$ | 1:1:1 | Dark Brown | 116 | 8.50 (8.93) | 24.37 (24.87) | 8.63 (9.04) | 762 (784) |
| - | $C_{34}H_{24}N_2O_2$ (MaL[2]) | - | - | - | Light Yellow | 82-84 | 5.25 (5.68) | - | - | 461 (493) |
| $PdCl_2$ (0.1819) | $C_{34}H_{24}N_2O_2$ (0.5053) | $C_6H_8N_2$ (0.1109) | $C_{40}H_{28}N_4Cl_2Pds$ | 1:1:1 | Light Brown | 113 | 7.11 (7.55) | 13.86 (14.34) | 9.13 (9.56) | 712 (742) |
| $PdCl_2$ (0.2189) | $C_{34}H_{24}N_2O_2$ (0.6081) | $C_5H_7N_3$ (0.1347) | $C_{39}H_{27}N_5Cl_2Pd$ | 1:1:1 | Brown | 125 | 9.04 (9.43) | 13.87 (14.32) | 9.94 (9.54) | 722 (743) |
| $PbCl_2$ (0.3782) | $C_{34}H_{24}N_2O_2$ (0.6698) | $C_6H_8N_2$ (0.1470) | $C_{40}H_{28}N_4Cl_2Pb$ | 1:1:1 | Light Brown | 137 | 6.22 (6.65) | 24.10 (24.58) | 7.94 (8.41) | 811 (843) |
| $PbCl_2$ (0.3986) | $C_{34}H_{24}N_2O_2$ (0.7059) | $C_5H_7N_3$ (0.1564) | $C_{39}H_{27}N_5Cl_2Pb$ | 1:1:1 | Dark Green | 126 | 7.90 (8.30) | 24.08 (24.55) | 7.96 (8.40) | 821 (844) |
| - | $C_{33}H_{23}N_3O_2$ (MaL[3]) | - | - | - | Green | 99 | 8.09 (8.51) | - | - | 469 (494) |
| $Pb(NO_3)_2$ (0.4209) | $C_{33}H_{23}N_3O_2$ (0.6273) | $C_6H_8N_2$ (0.1374) | $C_{39}H_{27}N_7O_6Pb$ | 1:1:1 | Reddish Brown | 131 | 10.51 (10.93) | 22.63 (23.10) | - | 872 (897) |
| $Pb(NO_3)_2$ (0.5220) | $C_{33}H_{23}N_3O_2$ (0.7779) | $C_5H_7N_3$ (0.1719) | $C_{38}H_{26}N_8O_6Pb$ | 1:1:1 | Dark Green | 118 | 12.86 (12.48) | 22.59 (23.07) | - | 868 (898) |

**IR Spectra**

The IR spectra of the ligands and their metal complexes were recorded as KBr pellets in the range 4000-200 $cm^{-1}$. The IR spectra of the complexes compare well the spectra of ligands and confirmed the formation of macrocyclic complexes with the proposed coordination pattern. The IR spectra of the ligands have bands due to ν(C=O) at 1665-1680 $cm^{-1}$. The spectra of all the complexes do not contain any $\nu(NH_2)$ and ν(C=O) vibrations, confirming that cyclization has occurred and characteristic bands of imine groups[18] ν(C=N) occur at 1625-1595 $cm^{-1}$. The remarkable changes observed for the IR spectra of the macrocyclic complexes are the absence of stretching and deformation vibrations of $-NH_2$ groups[19] indicating the deprotonation. The phenyl ring absorption appear in the 1465-1495 and 1355-1390 $cm^{-1}$ regions are assigned to $\nu_{asym}$ $C_6H_5$ and $\nu_{sym}$ $C_6H_5$, respectively. The spectra do not show any changes in the pyridine ring vibrations and it appears that in these complexes the nitrogen atom of pyridine does not participate in coordination. Strong and sharp bands for C-H stretching and bending vibrations appear at *ca* 2816 and 1408 $cm^{-1}$, respectively[18]. The presence of an aromatic C-N band in the complexes confirmed at 835 $cm^{-1}$.

It is evident from the spectra that both the amino groups of diamines react with oxygen atom of benzil, forming a two-carbon atom bridge between the two amino groups, similar to those reported in coordinated amines. The far infra-red spectra of Pd(II), Pt(II) and Pb(II) complexes showed absorption at 395-415, 430-455 and 410-440 $cm^{-1}$ due to ν(Pd-N), ν(Pt-N) and ν(Pb-N) vibrations[20], respectively, which are absent in the spectra of the corresponding ligands. Appearance of these bands unequivocally support the coordination of ligand molecule to the central metal atom. Further, appearance of new bands in the regions 1240, 982 and 870 $cm^{-1}$ are in agreement with the monodentate nature of the nitrato group[21].

**$^1H$ NMR Spectra**

The bonding pattern in the resulting complexes has been further substantiated by the proton magnetic resonance spectra of ligands and their respective metal derivatives. In the $^1H$ NMR spectra of the ligand $MaL^1$ and its metal complexes, a singlet observed at δ 3.30-3.45 ppm may be assigned to methylene protons adjacent to the nitrogen atom. The shift of the signal towards lower field is an indication of the coordination of the macrocycles. The spectrum of the ligand does not show any signal corresponding to the primary amino protons indicating that the proposed macrocyclic skeleton has been formed. The complex pattern of the aromatic protons were observed at δ 7.25 – 8.30 ppm in the spectra of ligands $MaL^1$, $MaL^2$ and $MaL^3$. A down field shifting in the position of this complex pattern strongly support the coordination of the azomethine nitrogens to the central metal.

**Electronic Spectra**

The electronic spectra of the ligand $MaL^1$ and its complexes were recorded in distilled DMSO. The maxima at 282-290 and 328-340 nm in the spectra of the metal complexes are due to π-π* electronic transitions. The position of these absorptions remain almost same as that of the ligand. However, a band appearing at 400 nm in the spectrum of the ligand, due to n-π* transitions within the >C=N chromophore, shows a shift due to the polarization in the >C=N band caused by the metal ligand electron interaction during the chelation. The shift of this band (*ca* 10nm) in the spectra of the complexes suggest the coordination of the nitrogen to the metal atom[22].

The electronic spectra of palladium (II) and platinum (II) complexes are also recorded. Three d-d-spin allowed transitions are expected in these complexes corresponding to the transitions from the three lower lying 'd' levels to the empty $d_{x^2-y^2}$ orbitals. The ground state is $^1A_{1g}$ and the excited states corresponding to the above transitions are $^1A_{2g}$, $^1B_{1g}$ and $^1E_g$ in order of increasing energy. Three d-d- bands are observed in the regions 545-555, 473-485 and 408-415 nm in the present palladium(II) complexes and 530-540, 448-469 and 386-398 nm in the case of platinum(II) complexes. These bands are attributed to $^1A_{1g} \rightarrow {}^1A_{2g}$, $^1A_{1g} \rightarrow {}^1B_{1g}$ and $^1A_{1g} \rightarrow {}^1E_g$ transitions, respectively[23,24]. The electronic spectra of these complexes indicate a square-planar geometry and the values obtained correspond to those reported earlier for the squar-planar complexes[24].

**$^{195}Pt$ NMR Spectra**

$^{195}Pt$ NMR spectra of the derivatives $[Pt(C_{35}H_{27}N_5)]Cl_2$ and $[Pt(C_{36}H_{28}N_4]Cl_2$ in DMSO have been recorded. These display single sharp peaks at δ-2482 ppm and -2501 ppm, indicative to the tetracoordinated state[25] of the metal in the said complexes.

**$^{13}C$ NMR Spectra**

The $^{13}C$ NMR spectra of the macrocyclic complexes are comparable to those of their ligands and the assigned peaks positions are listed in Table 2. The shifting in the positions of carbon resonances attached to the azomethine nitrogen indicate the involvement of these atoms in coordination and support the formation of macrocyclic framework, as reported earlier also[26-31].

**X-Ray Powder Diffraction Studies**

The possible geometry of the finely powdered products $[Pd(C_{39}H_{27}N_5)]Cl_2$ and $[Pt(C_{35}H_{27}N_5)]Cl_2$ have been deduced on the basis of X-ray powder diffraction studies. The observed interplanar spacing values ('d' in A°), have been measured from the diffractogram of these compounds and the Miller indices h, k and l have been assigned to each d value and 2θ angles are reported in Tables 3 and 4. The results show that both the compounds belong to 'orthorhombic' crystal system having unit cell parameters as :

a = 25.761, b = 17.535, c = 10.257

max. dev. of 2θ = 0.2 ; α = β = γ = 90°

and a = 25.775, b = 17.542, c = 10.297

max. dev. of 2θ = 0.1 ; α = β = γ = 90°, respectively.

**Table 2. [13]C NMR Spectral Data (δ, ppm) of Ligand and their Corresponding Metal Complexes.**

| Compound | Chemical Shift Values | | | | R | | Aromatic Carbons | | | |
|---|---|---|---|---|---|---|---|---|---|---|
| | >C=O | >C=N | -CH2-N< | >C-N | $C_b$ | $C_c$ | $C_1$ | $C_2$ | $C_3$ | $C_4$ |
| $C_{30}H_{24}N_2O_2$ (MaL[1]) | 176.62 | 160.92 | 42.37 | - | - | - | 128.61 | 127.20 | 126.77 | 126.12 |
| $C_{36}H_{28}N_4Cl_2Pt$ | - | 157.89 | 46.19 | 143.09 | 133.58 | 135.79 | 129.56 | 128.41 | 127.85 | 127.50 |
| $C_{35}H_{27}N_5Cl_2Pt$ | - | 156.93 | 44.77 | 142.51 | 132.91 | 136.53 | 128.90 | 128.68 | 126.23 | 125.94 |
| $C_{34}H_{24}N_2O_2$ (MaL[2]) | 175.52 | 157.22 | - | 143.67 | 134.50 | 136.68 | 127.67 | 127.28 | 126.51 | 125.79 |
| $C_{40}H_{28}N_4Cl_2Pd$ | - | 153.83 | - | 141.76 | 132.04 | 139.66 | 128.81 | 128.10 | 127.60 | 126.72 |
| $C_{39}H_{27}N_5Cl_2Pd$ | - | 153.40 | - | 142.10 | 132.34 | 139.17 | 129.16 | 128.55 | 128.11 | 127.49 |
| $C_{40}H_{28}N_4Cl_2Pb$ | - | 152.43 | - | 141.20 | 131.08 | 138.06 | 129.41 | 129.17 | 128.79 | 128.66 |
| $C_{39}H_{27}N_5Cl_2Pb$ | - | 152.36 | - | 141.47 | 131.95 | 138.33 | 128.75 | 128.40 | 127.78 | 127.49 |
| $C_{33}H_{23}N_3O_2$ (MaL[3]) | 174.48 | 156.72 | - | 142.87 | 131.21 | 135.30 | 127.16 | 126.60 | 126.24 | 125.32 |
| $C_{39}H_{27}N_7O_6Pb$ | - | 152.49 | - | 140.28 | 133.98 | 138.16 | 129.66 | 129.20 | 129.15 | 128.91 |
| $C_{38}H_{26}N_8O_6Pb$ | - | 151.86 | - | 140.94 | 132.55 | 136.56 | 129.91 | 129.33 | 128.74 | 128.12 |

**Table 3. X-Ray Powder Diffraction Data for the Compound $[Pd(C_{39}H_{27}N_5)]Cl_2$**

| S. No. | 2θ (Obs.) | 2θ (Calcd.) | Delta | h | k | l | d-Spacing (Obs.) A° |
|---|---|---|---|---|---|---|---|
| 1. | 13.80 | 13.87 | -0.07 | 2 | 0 | 1 | 8.063 |
| 2. | 18.40 | 18.44 | -0.04 | 4 | 1 | 0 | 6.059 |
| 3. | 18.92 | 18.84 | 0.08 | 2 | 2 | 1 | 5.894 |
| 4. | 22.20 | 22.21 | -0.01 | 1 | 0 | 2 | 5.031 |
| 5. | 24.40 | 24.32 | 0.08 | 2 | 1 | 2 | 4.584 |
| 6. | 26.40 | 26.22 | 0.18 | 3 | 1 | 2 | 4.242 |
| 7. | 27.60 | 27.50 | 0.10 | 5 | 2 | 1 | 4.061 |
| 8. | 28.30 | 28.31 | -0.01 | 6 | 0 | 1 | 3.962 |
| 9. | 28.92 | 29.04 | -0.12 | 6 | 1 | 1 | 3.879 |
| 10. | 30.48 | 30.52 | -0.04 | 7 | 0 | 0 | 3.685 |
| 11. | 31.20 | 31.20 | -0.00 | 7 | 1 | 0 | 3.602 |
| 12. | 31.80 | 31.98 | -0.18 | 3 | 3 | 2 | 3.536 |
| 13. | 32.60 | 32.51 | 0.09 | 6 | 3 | 0 | 3.451 |
| 14. | 34.20 | 34.22 | -0.02 | 6 | 0 | 2 | 3.294 |
| 15. | 34.60 | 34.71 | -0.11 | 2 | 1 | 3 | 3.257 |
| 16. | 36.40 | 36.47 | -0.07 | 3 | 5 | 1 | 3.101 |
| 17. | 41.80 | 41.79 | 0.01 | 6 | 5 | 0 | 2.715 |

Refined value of a = 25.761, b = 17.535, c = 10.257 (Orthorhombic system)

**Table 4. X-Ray Powder Diffraction Data for the Compound $[Pt(C_{35}H_{27}N_5)]Cl_2$**

| S. No. | 2θ (Obs.) | 2θ (Calcd.) | Delta | h | k | l | d-Spacing (Obs.) A° |
|---|---|---|---|---|---|---|---|
| 1. | 15.36 | 15.35 | 0.00 | 2 | 2 | 0 | 7.250 |
| 2. | 16.91 | 16.89 | 0.02 | 3 | 0 | 1 | 6.590 |
| 3. | 22.62 | 22.59 | 0.03 | 5 | 1 | 0 | 4.940 |
| 4. | 25.85 | 25.85 | 0.00 | 4 | 3 | 0 | 4.330 |
| 5. | 27.47 | 27.47 | 0.00 | 5 | 2 | 1 | 4.080 |
| 6. | 30.78 | 30.76 | 0.02 | 3 | 4 | 1 | 3.650 |
| 7. | 31.95 | 31.91 | 0.04 | 3 | 3 | 2 | 3.520 |
| 8. | 37.94 | 37.91 | 0.03 | 7 | 3 | 1 | 2.980 |
| 9. | 42.21 | 42.22 | -0.00 | 4 | 3 | 3 | 2.690 |
| 10. | 44.65 | 44.65 | 0.00 | 10 | 1 | 0 | 2.550 |
| 11. | 46.38 | 46.38 | -0.00 | 2 | 7 | 0 | 2.460 |
| 12. | 48.25 | 48.18 | 0.07 | 8 | 5 | 0 | 2.370 |

Refined value of a = 25.775, b = 17.542, c = 10.297 (Orthorhombic system)

**$^{207}$Pb NMR Spectra**

$^{207}$Pb Chemical shift values for $C_{40}H_{28}N_4Cl_2Pb$, $C_{39}H_{27}N_5Cl_2Pb$, $C_{39}H_{27}N_7O_6Pb$ and $C_{38}H_{26}N_8O_6Pb$ have been observed at δ 2225, 2203, 2197 and 2186 ppm, respectively, which indicated a hexacoordinated environment around the lead atom. These results are found to be in accordance with the results obtained by West et.al[32] and Godwin et.al[33].

Thus, on the basis of the above spectral and analytical data, following structures (*1*) and (*2*) for palladium, platinum and lead macrocyclic complexes have been proposed.

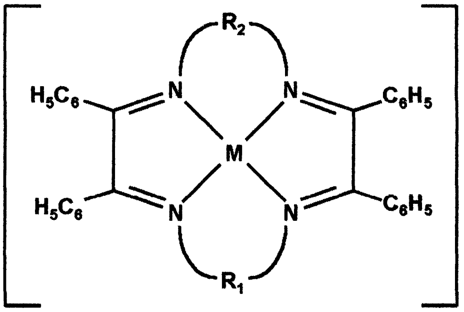

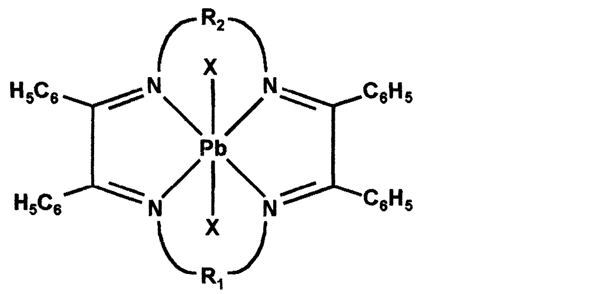

*(1)* Where, M = Pd$^{II}$ or Pt$^{II}$

*(2)* X = Cl or $NO_3$

$R_2$ = 1,2-$C_6H_4$ or 2,6-$C_5H_3N$

### BIOLOGICAL STUDIES

Biochemical applications have greater demand now-a-days. Activities of fungi and bacteria on several compounds give more important information about complexes. So it is promoted us to screen all the macrocyclic complexes and ligands to find out which part of the molecule is actually responsible for its physiological activity.

**Antifungal Activity**

The antifungal activity of ligands MaL$^1$, MaL$^2$ and MaL$^3$, alongwith that of their corresponding palladium(II), platinum(II) and lead(II) complexes, has been evaluated against *Fusarium oxysporum*, and *Macrophomina phaseolina* by the agar plate technique[34]. The compounds were directly mixed with the medium in different concentrations. Controls were also run and three replicates were used in each case. The linear growth of the fungus was obtained by measuring the diameter of the fungal colony after 96 hours (Table 5). The percentage inhibition in all of the replicates was calculated by the equation :

$$\text{Percentage inhibition} = \frac{(C-T) \times 100}{C}$$

Where C is the diameter of the fungal colony in the control plate and T is the diameter of the fungal colony in the test plate.

**Table 5. Fungicidal Screening Data of the Ligands and their Corresponding Metal Complexes.**

| Compound | Average percentage inhibition after 96 hours (conc. in ppm) | | | | | | | |
|---|---|---|---|---|---|---|---|---|
| | *Fusarium oxysporum* | | | | *Macrophomina phaseolina* | | | |
| | 25 | 50 | 100 | 200 | 25 | 50 | 100 | 200 |
| $C_{30}H_{24}N_2O_2$ | 17 | 26 | 35 | 44 | 19 | 31 | 39 | 48 |
| $C_{36}H_{28}N_4Cl_2Pt$ | 25 | 38 | 48 | 55 | 34 | 45 | 60 | 62 |
| $C_{35}H_{27}N_5Cl_2Pt$ | 26 | 40 | 49 | 55 | 35 | 46 | 60 | 64 |
| $C_{34}H_{24}N_2O_2$ | 22 | 35 | 42 | 49 | 23 | 37 | 43 | 50 |
| $C_{40}H_{28}N_4Cl_2Pd$ | 26.5 | 41 | 46 | 56 | 27 | 43 | 49 | 58 |
| $C_{39}H_{27}N_5Cl_2Pd$ | 28 | 42 | 45 | 58 | 30 | 44 | 46 | 60 |
| $C_{40}H_{28}N_4Cl_2Pb$ | 37 | 50 | 60 | 68 | 40 | 52 | 61 | 71 |
| $C_{39}H_{27}N_5Cl_2Pb$ | 38.5 | 51 | 62 | 70 | 41 | 52 | 63 | 72 |
| $C_{33}H_{23}N_3O_2$ | 24 | 41 | 46 | 52 | 30 | 44 | 50 | 56 |
| $C_{39}H_{27}N_7O_6Pb$ | 42.5 | 53 | 65 | 71 | 44 | 54 | 67 | 73 |
| $C_{38}H_{26}N_8O_6Pb$ | 43 | 52 | 66 | 72 | 45 | 56 | 69 | 75 |
| Standard (Bavistin) | 83 | 86 | 100 | 100 | 80 | 82 | 100 | 100 |

**Antibacterial Activity**

The antibacterial activity of the ligands MaL$^1$, MaL$^2$ and MaL$^3$ and their corresponding palladium(II), platinum(II) and lead(II) complexes has also been tested against *Escherichia coli*, *Xanthomonas campestris* and *Staphylococcus aureus* by the inhibition zone technique[35]. All the compounds were dissolved in methanol in 500 and 1000 ppm concentrations. Paper discs of Whatman No.1 paper with a diameter of 5mm, were soaked in these solutions. These discs were placed on the appropriate medium previously seeded with organism in Petri dishes and stored in an incubator at 29 ± 1°C. The inhibition zone thus formed around each disc containing the test compound was measured (in mm) after 24 hours. The results of these studies are shown in Table 6.

**Table 6. Bactericidal Screening Data of the Ligands and their Corresponding Metal Complexes.**

| Compound | Diameter of inhibition zone (mm) after 24 hours (conc. in ppm) | | | | | |
|---|---|---|---|---|---|---|
| | *Escherichia coli* | | *Xanthomonas campestris* | | *Staphylococcus aureus* | |
| | 500 | 1000 | 500 | 1000 | 500 | 1000 |
| $C_{30}H_{24}N_2O_2$ | 4 | 6 | 5 | 6 | 6 | 8 |
| $C_{36}H_{28}N_4Cl_2Pt$ | 6 | 8 | 7 | 9 | 8 | 10 |
| $C_{35}H_{27}N_5Cl_2Pt$ | 7 | 8 | 6 | 8 | 9 | 10 |
| $C_{34}H_{24}N_2O_2$ | 5 | 7 | 5 | 8 | 7 | 10 |
| $C_{40}H_{28}N_4Cl_2Pd$ | 7 | 8 | 7 | 9 | 9 | 12 |
| $C_{39}H_{27}N_5Cl_2Pd$ | 8 | 10 | 8 | 11 | 10 | 12 |
| $C_{40}H_{28}N_4Cl_2Pb$ | 12 | 15 | 8 | 12 | 13 | 14 |
| $C_{39}H_{27}N_5Cl_2Pb$ | 13 | 15 | 10 | 11 | 12 | 13 |
| $C_{33}H_{23}N_3O_2$ | 6 | 9 | 4 | 6 | 6 | 11 |
| $C_{39}H_{27}N_7O_6Pb$ | 9 | 12 | 7 | 10 | 10 | 14 |
| $C_{38}H_{26}N_8O_6Pb$ | 10 | 11 | 8 | 10 | 11 | 12 |
| Standard (Streptomycin) | 17 | 18 | 3 | 5 | 15 | 17 |

The above mentioned observations reveal that there is an increase in the toxicity of the complexes as compared to the ligands and the inhibition of the growth of the microorganisms was found to be dependent on the concentration of the complexes. The toxicity increases as the concentration and complexation increases. The results recorded from the biological activity were also further compared with the standard fungicide *Bavistin* and conventional bactericide *Streptomycin*. The mechanism of the toxicity of these complexes to micro-organisms may be due to the inhibition of energy or ATP production[36], for instance by inhibition of respiration or by uncoupling of oxidative phosphorylation. The energy producing processes are located partly in the cytoplasm and partly in the mitochondria. On comparing the influence of the metal ion on the intrinsic fungitoxicity of the metal chelates, it has been inferred that lead (II) complexes are more active as compared with palladium (II) and platinum (II). The activity of palladium(II) and platinum(II) complexes are more or less of the same order. According to Lawrence et al.[37] the biocidal properties of the complexes against various microorganisms depend on the impermeability of the cell. The bactericidal activity of complexes was greater towards gram positive strains as compared to the gram negative strains.

**Antifertility Activity**

Male rats obtained from ICMR, New Delhi, India, were used. Colony bred, sexually mature sprague dawley albino rats (***Rattus norvegicus***) were housed in clean wiremesh cages and kept under controlled laboratory conditions of temperature and illumination (12 h light/12 h dark cycle; 25 ± 3°C, 35-60% humidity) and provided with a standard pellet diet (Lipton India Ltd.) and water ***ad libitum***. The male rats proven fertility were divided into different groups containing equal number of animals. One group served with vehicle (olive oil) was treated as control. In other groups, starting material, ligands and their complexes (50 mg/kg body weight) suspended in 0.2 ml. olive oil, were given orally for a period of 60 days. At the end of experimental period, the animals were screened for fertility test and autopsized for the study of histological and biochemical changes. Reproductive organs were dissected out, cleaned of adhesing tissues and blood, weighed and kept at –20°C for biochemical estimations. The testes were also fixed in Bouin's fluid for histological examination.

The sperm motility and density of cauda epididymal spermatozoa[38] , the total cholesterol[39] , sialic acid[40] and fructose[41] were determined by the standard laboratory techniques.

The testicular morphology, testicular sperm density, sperm motility, density of cauda epididymal spermatozoa and fertility in mating trails and biochemical parameters of reproductive organs with $MaL^2$, $MaL^3$ and their lead complexes ***in vivo*** have been examined and discussed (Tables 7-11). The results show that the ligands alone were able to inhibit fertility but due to added synergistic effects of lead(II) complexes, their activity got enhanced.

**Evaluation of Male Fertility Potential**

At the termination of experimentation, males from each group were cohabited individually with proestrous females in the ratio of 1:3. Successful mating in each case was confirmed by the presence of a copulation plug and spermatozoa in the vaginal smear. The day when spermatozoa were detected in the smear was designated as day zero of pregnancy. Such females were laprotomized on day 15 *post coitum* and the number of implantation sites, if any, in the uteri were recorded[42] .

In the present study we investigated a variety of male reproductives and points following acute exposure to ligands as well as their metal complexes and the results have been described under the following headings :

**Body and Genital Organ Weights**

No significant change in the body weights of rats was observed in any experimental group when compared with their initial body weights, after treatment with ligands and their complexes. However, the weights of testes, epididymis, seminal vesicle and ventral prostate were decreased significantly in ligands and metal derivatives treated rats, which was probably due to the demage of the spermatogonial cells. (Table 7).

**Table 7. Alteration of Percent Organ Weight of the Genital Organs of Male Rat treated with Ligands and their Corresponding Complexes.**

| Group | Treatment | Body Weight (g) | | Testes mg/100g b.wt. | Epididymis mg/100 g b.wt. | Seminal Vesicle mg/100g b.wt. | Ventral Prostate mg/100g b.wt. |
|---|---|---|---|---|---|---|---|
| | | Initial | Final | | | | |
| A | Control | $190 \pm 10$ | $208 \pm 9^{d}$ | $1250 \pm 80$ | $470 \pm 40$ | $380 \pm 25$ | $270 \pm 22$ |
| B | $PbCl_2$ | $180 \pm 8$ | $192 \pm 10^{d}$ | $925 \pm 35^{b}$ | $300 \pm 30^{b}$ | $305 \pm 10^{b}$ | $200 \pm 20^{b}$ |
| C | $C_{34}H_{24}N_2O_2$ ($MaL^2$) | $193 \pm 9$ | $210 \pm 6^{d}$ | $1010 \pm 50^{a}$ | $325 \pm 40^{a}$ | $315 \pm 20^{a}$ | $225 \pm 15^{a}$ |
| D | $C_{40}H_{28}N_4Cl_2Pb$ | $198 \pm 7$ | $215 \pm 8^{d}$ | $640 \pm 70^{c}$ | $205 \pm 10^{c}$ | $198 \pm 25^{c}$ | $160 \pm 30^{c}$ |
| E | $Pb(NO_3)_2$ | $175 \pm 12$ | $189 \pm 8^{d}$ | $900 \pm 50^{b}$ | $310 \pm 50^{b}$ | $302 \pm 18^{b}$ | $205 \pm 18^{b}$ |
| F | $C_{33}H_{23}N_3O_2$ ($MaL^3$) | $182 \pm 5$ | $200 \pm 7^{d}$ | $990 \pm 80^{a}$ | $340 \pm 30^{a}$ | $328 \pm 10^{a}$ | $230 \pm 10^{a}$ |
| G | $C_{39}H_{27}N_7O_6Pb$ | $178 \pm 11$ | $190 \pm 5^{d}$ | $620 \pm 50^{c}$ | $180 \pm 35^{c}$ | $200 \pm 19^{c}$ | $170 \pm 25^{c}$ |

**Sperm Motility and Sperm Density**

A significant decline in sperm motility in cauda epididymis was observed in animals after treatment with both the ligands and their compounds (Table 8).

**Table 8. Sperm Dynamics after Treatment with Ligands and their Corresponding Complexes.**

| Group | Treatment | Sperm Motility (%) Cauda Epididymis | Sperm Density (million/ml) | |
|---|---|---|---|---|
| | | | Testes | Cauda epididymis |
| A | Control | $79 \pm 5.0$ | $3.9 \pm 0.25$ | $59 \pm 5$ |
| B | $PbCl_2$ | $62 \pm 8^{b}$ | $2.9 \pm 0.35^{b}$ | $42 \pm 3^{b}$ |
| C | $C_{34}H_{24}N_2O_2$ ($MaL^2$) | $66 \pm 4.5^{a}$ | $3.2 \pm 0.20^{a}$ | $45 \pm 4^{a}$ |
| D | $C_{40}H_{28}N_4Cl_2Pb$ | $51 \pm 4^{c}$ | $1.8 \pm 0.32^{c}$ | $25 \pm 7^{c}$ |
| E | $Pb(NO_3)_2$ | $65.5 \pm 2^{b}$ | $2.8 \pm 0.5^{b}$ | $41 \pm 4^{b}$ |
| F | $C_{33}H_{23}N_3O_2$ ($MaL^3$) | $67 \pm 3^{a}$ | $3.3 \pm 0.1^{a}$ | $44 \pm 3^{a}$ |
| G | $C_{39}H_{27}N_7O_6Pb$ | $48 \pm 5^{c}$ | $1.6 \pm 0.4^{c}$ | $20 \pm 3^{c}$ |

**Biochemical Parameters of Reproductive Organs**

**Sialic acid :** Sialic acid contents of testes, epididymis, seminal vesicle and ventral prostate reduced significantly after treatment with ligands and their complexes (Table 9). The reduction in sialic acid levels is probably due to the inhibition of spermatogenesis in testes.

**Table 9. Changes in Sialic Acid contents of Genital Organs after Treatment with Ligands and their Corresponding Complexes.**

| **Group** | Treatment | Sialic Acid (mg/gm) | | | |
|---|---|---|---|---|---|
| | | **Testes** | Epididymis | Seminal Vesicles | **Ventral Prostate** |
| A | Control | $8.9 \pm 0.8$ | $7.5 \pm 0.9$ | $8.2 \pm 0.7$ | $8.4 \pm 0.5$ |
| B | $PbCl_2$ | $6.9 \pm 0.9^{a}$ | $5.9 \pm 0.8^{a}$ | $6.6 \pm 0.8^{a}$ | $6.7 \pm 0.6^{a}$ |
| C | $C_{34}H_{24}N_2O_2$ ($MaL^2$) | $7.1 \pm 0.8^{a}$ | $5.7 \pm 0.7^{a}$ | $7.0 \pm 0.2^{a}$ | $6.9 \pm 0.5^{a}$ |
| D | $C_{40}H_{28}N_4Cl_2Pb$ | $4.3 \pm 0.5^{c}$ | $3.8 \pm 0.5^{c}$ | $3.5 \pm 0.8^{c}$ | $4.3 \pm 0.6^{c}$ |
| E | $Pb(NO_3)_2$ | $6.0 \pm 0.7^{b}$ | $5.4 \pm 1.1^{b}$ | $6.0 \pm 1.0^{b}$ | $6.4 \pm 0.7^{a}$ |
| F | $C_{33}H_{23}N_3O_2$ ($MaL^3$) | $6.8 \pm 0.8^{a}$ | $5.3 \pm 1.0^{b}$ | $6.7 \pm 0.6^{a}$ | $6.5 \pm 0.4^{a}$ |
| G | $C_{39}H_{27}N_7O_6Pb$ | $4.1 \pm 0.3^{c}$ | $3.3 \pm 0.2^{c}$ | $3.2 \pm 0.9^{c}$ | $4.1 \pm 0.3$ |

**Testicular Cholesterol and Glycogen :** Total cholesterol and glycogen contents of testes were reduced after the treatment with various compounds (Table 10).

**Table 10. Changes in Tissue Cholesterol, Glycogen and Fructose Levels after Treatment with Ligands and their Corresponding Complexes.**

| Group | Treatment | Cholesterol (mg/gm) Testes | Fructose (mg/gm) Seminal Vesicle | Glycogen (mg/gm) Testes |
|---|---|---|---|---|
| A | Control | 8.5 ± 0.7 | 470 ± 20 | 5.7 ± 0.5 |
| B | $PbCl_2$ | 6.6 ± $0.5^b$ | 390 ± $25^b$ | 4.3 ± $0.5^b$ |
| C | $C_{34}H_{24}N_2O_2$ ($MaL^2$) | 7.0 ± $0.5^a$ | 410 ± $25^a$ | 4.2 ± $0.6^b$ |
| D | $C_{40}H_{28}N_4Cl_2Pb$ | 3.5 ± $0.4^c$ | 210 ± $20^c$ | 3.1 ± $0.3^c$ |
| E | $Pb(NO_3)_2$ | 6.9 ± $0.5^a$ | 415 ± $15^a$ | 4.4 ± $0.4^b$ |
| F | $C_{33}H_{23}N_3O_2$ ($MaL^3$) | 6.8 ± $0.6^a$ | 420 ± $10^a$ | 4.1 ± $0.8^b$ |
| G | $C_{39}H_{27}N_7O_6Pb$ | 3.2 ± 0.3 | 205 ± $18^c$ | 2.9 ± $0.4^c$ |

**Fructose :** Fructose contents of seminal vesicle declined significantly in all experimental groups (Table 10).
**Phosphatases :** Acid and alkaline phosphate contents of various reproductive tissues were declined significantly in all experimental groups (Table 11).

**Table 11. Alteration in Acid and Alkaline Phosphatase Enzyme Activity of Male Genital Organs after Treatment with Ligands and their Corresponding Complexes.**

| **Group** | Treatment | Acid Phosphatase (Bodansky unit = mg Pi/g/hr) | | | |
|---|---|---|---|---|---|
| | | Testes | Epididymis | Seminal Vesicle | Ventral Prostate |
| A | Control | 3.48± 0.05 | 2.9± 0.06 | 1.2± 0.05 | 1.9± 0.08 |
| B | $PbCl_2$ | 2.0± $0.6^b$ | 2.1± $0.03^a$ | 0.8± $0.02^b$ | 1.1± $0.05^b$ |
| C | $C_{34}H_{24}N_2O_2$ ($MaL^2$) | 2.5± $0.4^a$ | 2.0± $0.03^a$ | 0.9± $0.05^a$ | 1.2± $0.03^b$ |
| D | $C_{40}H_{28}N_4Cl_2Pb$ | 1.5± $0.1^c$ | 1.1± $0.05^c$ | 0.6± $0.02^c$ | 0.5± $0.04^c$ |
| E | $Pb(NO_3)_2$ | 2.2± $0.3^b$ | 1.9± $0.19^a$ | 0.9± $0.06^a$ | 1.2± $0.09^b$ |
| F | $C_{33}H_{23}N_3O_2$ ($MaL^3$) | 2.4± $0.4^a$ | 1.8± $0.18^a$ | 0.8± $0.1^a$ | 1.3± $0.09^b$ |
| G | $C_{39}H_{27}N_7O_6Pb$ | 1.3± $0.2^c$ | 1.2± $0.06^c$ | 0.4± $0.07^c$ | 0.4± $0.05^c$ |

| **Group** | Alkaline Phosphatase (Bodansky unit = mg Pi/g/hr.) | | | |
|---|---|---|---|---|
| | Testes | Epididymis | Seminal Vesicle | Ventral Prostate |
| A | 2.0± 0.1 | 5.9± 0.4 | 3.9± 0.4 | 3.8± 0.2 |
| B | 1.5± $0.05^b$ | 4.5± $0.5^a$ | 2.5± $0.1^b$ | 2.9± $0.2^b$ |
| C | 1.6± $0.08^a$ | 4.8± $0.3^a$ | 2.8± $0.1^a$ | 3.0± $0.3^a$ |
| D | 0.6± $0.05^c$ | 2.1± $0.4^c$ | 1.9± $0.2^c$ | 1.1± $0.2^c$ |
| E | 1.4± $0.08^b$ | 4.7± $0.1^a$ | 2.6± $0.2^a$ | 2.8± $0.4^b$ |
| F | 1.3± $0.05^b$ | 4.6± $0.1^a$ | 2.7± $0.2^a$ | 2.7± $0.5^b$ |
| G | 0.5± $0.07^c$ | 3.1± $0.2^c$ | 1.5± $0.1^c$ | 1.3± $0.2^c$ |

The study revealed that treatment with $PbCl_2$, $Pb(NO_3)_2$, ligands and their complexes resulted in a significant reduction in the weights of testes, epididymis, seminal vesicle and ventral prostate. This may be due to the altered level of circulating androgens which adversely affects the structure, size and functional integrity of the organs of reproduction[43,44]. This contention was also supported by a consequent reduction in motility in cauda epididymis and sperm density in testes and cauda epididymis[45]. It has been reported that lead acetate administration to male rats caused low production of testicular testosterone and structural alteration in Leyding cell.

In the present studies reduction in total cholesterol, fructose, glycogen contents and alteration in the contents of androgen-sensitive enzymes, viz., acid and alkaline phosphatase[46] in the male reproductive tissues of experimental rats, is also indicative of impairement of the functional integrity of the genital organs. Further, a significant reduction in sialic acid contents of male reproductive organs after treatment with $PbCl_2$, $Pb(NO_3)_2$, ligand and their complexes can also be related to the antiandrogenic nature of the test substance since the concentration of sialic acid is dependent upon androgen production[47]. Reduction in the fructose concentration of seminal vesicle after treatment of ligands and complexes further support the antiandrogenic nature of these compounds. The results of the present study establish that the $PbCl_2$, $Pb(NO_3)_2$ and their complexes are more effective than their ligands, i.e., $MaL^2$ and $MaL^3$ in regulating fertility.

### ACKNOWLEDGEMENT

The authors are thankful to Government of India, Ministry of Science and Technology, New Delhi, for financial support and Dr. R.D. Singh for help in getting recorded the Pb-NMR spectra of our compounds.

### REFERENCES

1. E. Blinn and D.H. Busch, *Inorg. Chem.*, **7**, 820 (1968).
2. L. Canali and D.C. Sherrington, *Chem. Soc. Rev.*, **28**, 85 (1999).
3. G.A. Melson, *Coordination Chemistry of Macrocyclic Compounds*. Plenum Press, New York, p.1 (1979).

4. Z. Shourong, L. Huakuan, X. Jingchun and C. Yunti, *Polyhedron*, **13**, 759 (1994).
5. W.E. Keyes, J.B.R. Dunn and T.M. Loehr, *J. Am. Chem. Soc.*, **99**, 5427 (1977).
6. Z.A. Siddiqi and V.J. Mathew, *Polyhedron*, **13**, 799 (1994).
7. N.S. Enikolopyan, K.A. Bogdanova and K.A. Askarov, *Russ. Chem. Rev.*, **52**, 13 (1983).
8. M.M. Taqui Khan, S.B. Halligudi and S.H.R. Abdi, *J. Mol. Catal.*, **44**, 179 (1988).
9. P.J. McCarthy, R.J. Hovey, K. Veno and A.E. Martell, *J. Am. Chem. Soc.*,, **77**, 5820 (1955).
10. Q. Lu, R.J. Motekaitis, J.H. Reibenspies and A.E. Martell, *Inorg. Chem.*, **34**, 4958 (1995).
11. A.I. Vogel, "*A Text-Book of Inorganic Analysis*", Longmans Green and Co., London (1968).
12. A.I. Vogel, "*Text-Book of Quantitative Inorganic Analysis*", Longmans Green, ELBS, London, p. 512 (1962).
13. A.I. Vogel, : "*A Text-Book of Practical Organic Chemistry*", 4th Edition, Longmans, ELBS, London, p. 232 (1978).
14. R. Hesse, *Acta. Cryst.*, **1**, 200 (1948).
15. H. Lipson, *Acta. Cryst.*, **2**, 43 (1949).
16. T. Ito, "*X-ray Studies on Polymorphism*" Maruzen Co. Ltd., Japan, p. 187 (1950).
17. H.K. Sharma, S. Chandra and S. Gupta, *Synth. React. Inorg. Met.-Org. Chem.*, **27**, 695 (1997).
18. N.B. Colthup, L.H. Dally and S.E. Wiberley, "*Introduction to Infrared and Raman Spectroscopy*" (Academic Press, New York), (1964).
19. M. Shakir, S.P. Varkey and O.S.M. Nasmon, *Indian J. Chem.*, **35A**, 671 (1996).
20. K.I. Goldberg, J. Valdes – Martinez, G. Espinosa – Perez, L.J. Ackerman, D.X. West, *Polyhedron*, **18**, 1177 (1999).
21. M. Shakir, S.P. Varkey and P.S. Hameed, *Polyhedron*, **13**, 1355 (1994).
22. K. Singh, P. Dixit, R.V. Singh and J.P. Tandon, *Main Group Met. Chem.*, **12**, 155 (1989).
23. R.V. Singh, K. Sharma and N. Fahmi, *Trans. Met. Chem.*, **24**, 562 (1999).
24. H.B. Gray and C.J. Ballhousen, *J. Am. Chem. Soc.*, **85**, 260 (1963).
25. A. Irving, K.R. Koch and M. Motoetoe, *Inorg. Chim. Acta*, **206**, 195 (1993).
26. K. Fujita, M. Ikeda, Y. Nakano, T. Konodo and T. Mitsudo, *J. Chem. Soc., Dalton Trans.*, 2908 (1998).
27. J. Eilmes and E. Sledziewska, *Bull. Acad. Pol. Sci. Ser. Sci. Chim.*, **26**, 441 (1978).
28. J.B. Stothers, "Carbon-13 NMR Spectroscopy", *Academic Press, New York*, (1972).
29. Y. Hung, L.Y. Martin, S.C. Jackels, A.M. Tait and D.H. Busch, *J. Am. Chem. Soc.*, **99** 4029 (1977).
30. N.W. Alcock, N. Herron and P. Moore, *J. Chem. Soc. Dalton Trans.*, 1282 (1978).
31. M.R. Reddy, K.M. Raju and K.H. Reddy, *Indian J. Chem.*, **35A**, 677 (1996).
32. G.D. Fallon, L. Spiccia, B.O. West and Q. Zhang, *Polyhedron*, **16**, 19 (1997).
33. J.G. Harsfall, *Bot. Rev.*, 419 (1945).
34. E.S. Claudia, M.A. ter Horst, C.E. Forde, C.L. Stern, M.K. Zart and H.A. Godwin, *Inorg. Chem.*, **39**, 1391 (2000).
35. H.H. Thornberry, *Phytopathology*, **40**, 419 (1950).
36. M. Shakir, S.P. Varkey and T.A. Khan, *Indian J. Chem.*, **34A**, 72 (1995).
37. P.G. Lawrence, P.L. Harold and O.G. Francis, *Antibiotic and Chemotherapy*, **5**, 1597 (1980).
38. M.R.N. Prasad, N.J. Chinoy and K.M. Kadam, *Fert. Steril.*, **23**, 186 (1972).
39. B.L. Oser, *In Howk's Physiological Chemisry*, 14th ed., New York, McGraw Hill, 246 (1965).
40. L. Warren, *J. Biol. Chem.*, **234**, 1971 (1959).
41. D. Foreman, L. Gaylor, E. Evans and C.A. Trella, *Anat. Biochem.*, **56**, 584 (1973).
42. *A Method for Examining the Effect of a Plant Extract Administration Orally on the Fertility of Male Rats*, WHO-Protocol MB-50 (1983).
43. M. Chaturvedi and V.P. Dixit, *J. Environ. Sci.*, **1**, 89 (1997).
44. P.C. Mali, Antifertility Activity of Euphorbia Nerrifolia L. root extract in Male Rats, *Indian J. Environ. Sci.*, **3(2)**, 185 (1999).
45. A. Thoreux-Monley, C. Le-Goascogne, D. Segretain, B. Jegon and G. Pinon Lataillade, Lead Affects Steroidozenesis in Rat Leydig Cells in vivo and in vitro, *Toxicology*, **103(1)**, 53 (1995).
46. N. Sharma and D. Jacob, Fertility Supression of the Male Mouse after Administration of Mint Leaf Extract. *Phytother. Res.*, **10**, 175 (1996).
47. L.N. Shandilya, L.S. Ramaswami and N. Shandilya, Sialic acid Concentration in Reproductive Organs, Pituitary Gland and Urine of the Indian Langur Monkey (Presbytis entellus entellus) *J. Endocrinol.*,**73**, 207 (1977).

**Received: May 4, 2001 – Accepted: May 14, 2001 –**
**Accepted in publishable format: June 25, 2001**

